# Long-term effects of mixed planting on arbuscular mycorrhizal fungal communities in the roots and soils of *Juglans mandshurica* plantations

**DOI:** 10.1186/s12866-020-01987-1

**Published:** 2020-10-12

**Authors:** Li Ji, Yan Zhang, Yuchun Yang, Lixue Yang, Na Yang, Depeng Zhang

**Affiliations:** 1 Key Laboratory of Sustainable Forest Ecosystem Management-Ministry of Education, School of Forestry, Northeast Forestry University, Harbin, 150040 P. R. China; 2 Jilin Academy of Forestry, Changchun, 130033 P.R. China; 3 ZEHO Waterfront Ecological Environment Management Co., Ltd, Beijing, 100084 P. R. China

**Keywords:** Mixed plantation, Arbuscular mycorrhizal fungi, *Juglans mandshurica*, *Larix gmelinii*, Illumina MiSeq sequencing

## Abstract

**Background:**

Establishing mixed plantations is an effective way to improve soil fertility and increase forest productivity. Arbuscular mycorrhizal (AM) fungi are obligate symbiotic fungi that can promote mineral nutrient absorption and regulate intraspecific and interspecific competition in plants. However, the effects of mixed plantations on the community structure and abundance of AM fungi are still unclear. Illumina MiSeq sequencing was used to investigate the AM fungal community in the roots and soils of pure and mixed plantations (*Juglans mandshurica* × *Larix gmelinii*). The objective of this study is to compare the differential responses of the root and rhizosphere soil AM fungal communities of *Juglans mandshurica* to long-term mixed plantation management.

**Results:**

*Glomus* and *Paraglomus* were the dominant genera in the root samples, accounting for more than 80% of the sequences. Compared with that in the pure plantation, the relative abundance of *Glomus* was higher in the mixed plantation. *Glomus*, *Diversispora* and *Paraglomus* accounted for more than 85% of the sequences in the soil samples. The relative abundances of *Diversispora* and an unidentified genus of Glomeromycetes were higher and lower in the pure plantation, respectively. The Root_P samples (the roots in the pure plantation) had the highest number of unique OTUs (operational taxonomic units), which belonged mainly to an unidentified genus of Glomeromycetes, *Paraglomus*, *Glomus* and *Acaulospora*. The number of unique OTUs detected in the soil was lower than that in the roots. In both the root and soil samples, the forest type did not have a significant effect on AM fungal diversity, but the Sobs value and the Shannon, Chao1 and Ace indices of AM fungi in the roots were significantly higher than those in the soil.

**Conclusions:**

Mixed forest management had little effect on the AM fungal community of *Juglans mandshurica* roots and significantly changed the community composition of the soil AM fungi, but not the diversity.

## Background

Arbuscular mycorrhizal (AM) fungi are widespread and form symbiotic associations with approximately 80% of terrestrial plant species [1]. AM fungi not only promote absorption of mineral nutrients (C, N, P) [2–4], provide resistance to environmental stress [5, 6] and regulate intraspecific and interspecific competition in plants [7] but also directly and indirectly improve soil structure and affect the circulation of matter and flows of energy in the ecosystem [8]. Some studies have found that host plants preferentially allocate carbohydrates to the more beneficial symbionts when providing photosynthetic products to AM fungi [9–11]; this preference/selection results in varying AM community richness, composition and diversity, which induces the unequal effects of AM fungi on the growth rates of different plant species [12].

Previous studies have indicated that AM fungal taxonomic groups differ in terms of the main propagule form with which they colonize new roots and the allocation of biomass to the compartments of roots and soil [13]. To a certain extent, the composition and community structure of AM fungi depends on their propagation form (spores, infected root segments, extensive extraradical mycelium), and the difference between the propagation of AM fungi in root (intraradical) and soil (extraradical) samples may be related to the location and density of AM fungal propagules [14]. In addition, host plant selection for AM fungi exhibited strong selection pressure for AM fungi in the root system [15]. Moreover, the response of different AM fungal groups to host plants or the rhizosphere microenvironment may also lead to host plant selection preferences for certain AM fungal species [16]. There are differences in the distribution patterns of root carbon and the pathways to secondary metabolites or secretions, and these differences may cause changes in soil environmental conditions [9, 17]. A recent study found that AM fungal structures in roots and soil have different responses to biotic and abiotic factors. The AM fungal community structure in roots is mainly affected by host plants and disturbances (grazing), while AM fungi in soil are greatly affected by environmental factors [18].

AM fungi can form a huge mycorrhizal network and connect individual plants within the community, which facilitates the transport of nutrient resources among plants [19]. Therefore, neighboring plants can affect the impact of host plants on AM fungi or form specific AM fungal communities when multiple plants are planted in combination in terrestrial ecosystems [12, 20]. Previous studies have found that the construction of AM fungal communities is influenced by the identity of adjacent plants, and these studies have mostly occurred in greenhouses and invasive systems [21]. However, it is not clear how the coexistence of multiple plants affects arbuscular mycorrhizal fungal communities in natural ecosystems, especially in forest ecosystems.

Forests play important roles in producing wood and fuel, controlling soil erosion and maintaining ecosystem functions [22]. Monocultures account for 80% of the total forest planting area in China. Establishing and managing a monoculture are easier than doing the same for a mixed plantation, but it will reduce the ecological function of the forest, and long-term monoculture planting will cause litter quality and soil fertility to decline as well as causing other problems (e.g., biodiversity loss, soil degradation) [23]. Mixed plantations are an effective way to improve soil fertility and increase forest productivity [22, 24]. Numerous studies have shown that rational mixed plantations can improve soil fertility [25], nutrient cycling [25], stand productivity [26, 27], and tree nutrient status and resistance to pests and diseases [28]. However, the effects of mixed planting vary among tree species [29, 30]. Therefore, comparing the differences in AM fungal communities in different plantation types is conducive to developing a profound understanding of the stimulation mechanism by which mixed plantations influence AM fungal communities.

*Larix gmelinii* (an ectomycorrhizal species) is a major fast-growing afforestation species in northern China [31]. *L. gmelinii* plantations have long had certain problems, such as biodiversity loss and soil degradation with the rapid development of larch monocultures [32], that seriously affect their sustainable management. *Juglans mandshurica* (an arbuscular mycorrhizal species) is an important timber tree species in northeastern China and has great economic value. It has been reported that the mixed management of *Juglans mandshurica* and *Larix gmelinii* can improve soil fertility and stand productivity [33]; however, until now, the synergistic mechanism of mixed management, especially the interactions between the host plants and the soil AM fungal community structure, is still obscure. For this purpose, we compared the AM fungal community composition, structure and diversity in the roots and soil of *Juglans mandshurica* in pure and mixed plantations to provide a theoretical basis for the mechanism by which temperate mixed plantations influence AM fungal communities. We hypothesized that (1) mixed forests have a significant effect on the AM fungal community structure and composition in the roots and soil of *Juglans mandshurica* and that there will be higher AM fungal diversity and richness in the mixed plantation than in the pure plantation and (2) in both forest types, the diversity and richness of AM fungi in the soil will be higher than those in the roots.

## Results

### Soil properties and AM fungal colonization

The soil pH and total phenol content were significantly higher in the pure plantation than in the mixed plantation (Table 1; *P* < 0.05). The NH_4_^+^-N content was significantly higher in the mixed plantation than in the pure plantation. Compared with those in the mixed plantation, the soil moisture, P, C/N ratio, N_mic_, CPh, WSPh and colonization were mainly lower in the pure plantation, although no significant differences were found (*P* > 0.05).

**Table 1:** Soil variables and AM fungal colonization in pure and mixed plantations

Environmental variables	Pure plantation	Mixed plantation
pH	5.89 ± 0.06a	5.63 ± 0.04b
Moisture (%)	84.60 ± 2.23a	91.83 ± 10.59a
C (g·kg^−1^)	105.97 ± 10.12a	105.02 ± 20.26a
N (g·kg^−1^)	9.36 ± 0.8a	9.07 ± 1.72a
P (g·kg^−1^)	1.24 ± 0.07a	1.29 ± 0.16a
C/N	11.31 ± 0.16a	11.56 ± 0.06a
C_mic_ (mg·kg^−1^)	2299.94 ± 142.23a	1590.32 ± 372.04a
N_mic_ (mg·kg^−1^)	351.32 ± 21.99a	402.67 ± 16.29a
NH_4_^+^-N (mg·kg^−1^)	6.01 ± 0.28b	8.20 ± 0.57a
NO_3_^−^-N (mg·kg^−1^)	31.73 ± 1.23a	28.62 ± 2.37a
TPh (mg·kg^−1^)	690.17 ± 8.54a	536.37 ± 37.86b
CPh (mg·kg^−1^)	188.21 ± 6.93a	230.47 ± 13.81a
WSPh (mg·kg^−1^)	4.37 ± 0.19a	4.63 ± 0.33a
Colonization (%)	95.56 ± 2.94a	96.70 ± 2.41a

*C*_*mic*_ soil microbial biomass carbon, *N*_*mic*_ soil microbial biomass nitrogen, *TPh* soil total phenol, *CPh* soil complex phenol, *WSPh* soil water-souble phenol

### Sequence summary

Across all soil and root samples analyzed, 190,433 quality AM fungal sequences were yielded by Illumina MiSeq sequencing, with 11,388 ~ 19,701 AM fungal sequences per sample (mean = 15,869). The average read lengths were 238 bp for the 18S rRNA gene regions and higher than 99% of Good’s coverage for the 18S rRNA gene regions. For all samples, five orders, nine families, ten genera and one hundred and thirty-nine OTUs were detected. The rarefaction curves of OTUs tended to approach the saturation plateau at 97% sequence similarity for all samples (Supplementary Figure S1), which indicated that the sequencing depth was adequate for assessing the diversity of the AM fungal communities in all samples.

### AM fungal community composition

**Fig. 1:**
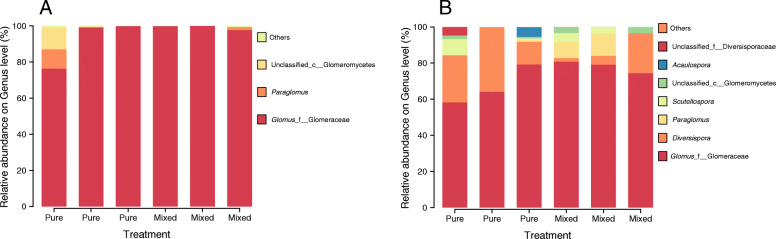
Relative abundances of main AM fungal genera in root (**a**) and soil (**b**) samples

**Fig. 2:**
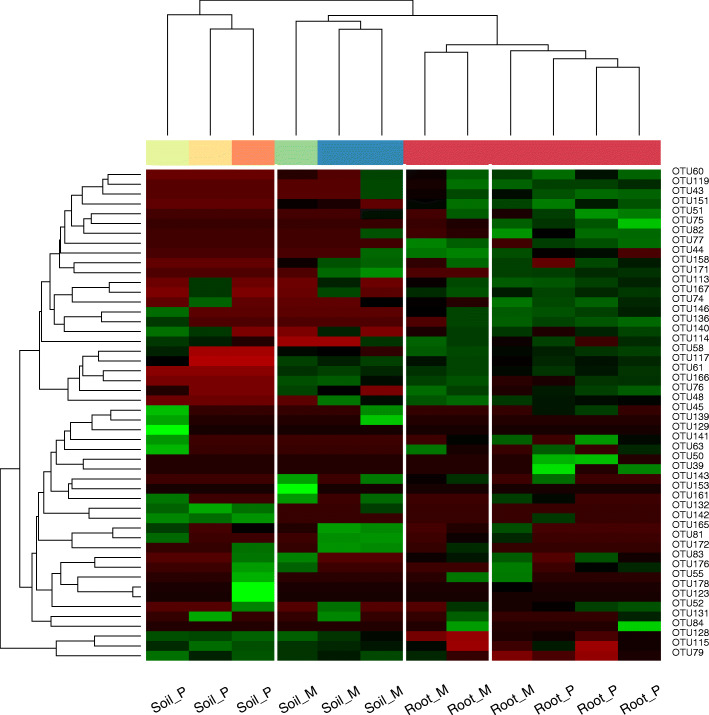
Heat map analyses of AM fungal communities in the root and soil samples. The relative abundances of the top 50 most abundant classified AM fungal OTUs were identified in each sample by colors deduced from the raw Z-scores. Hierarchical clustering of all samples was performed using average clustering method with the Euclidean distances. Soil_P represents soil sample in pure plantation; Soil_M represents soil sample in mixed plantation; Root_P represents root sample in pure plantation; Root_M represents root sample in mixed plantation

Across all root samples, nine AM fungal genera were detected. *Glomus* and *Paraglomus* were the dominant genera, accounting for more than 80% of the sequences (Fig. 1a). Compared with that in the pure plantation, the relative abundance of *Glomus* in root samples was higher in the mixed plantation (Fig. 1a). Across all soil samples, ten AM fungal genera were detected. *Glomus*, *Diversispora* and *Paraglomus* accounted for more than 85% of the sequences (Fig. 1b). The relative abundances of *Diversispora* and an unidentified genus of Glomeromycetes were higher and lower in the pure plantation, respectively. At the OTU level, 178 AM fungal taxa were detected. OTU79 and OTU76 were the dominant OTUs and had mean relative abundances of 12.15 and 6.54%, respectively (Supplementary Table S2, Fig. 2).

### AM fungal community structure and diversity

**Fig. 3:**
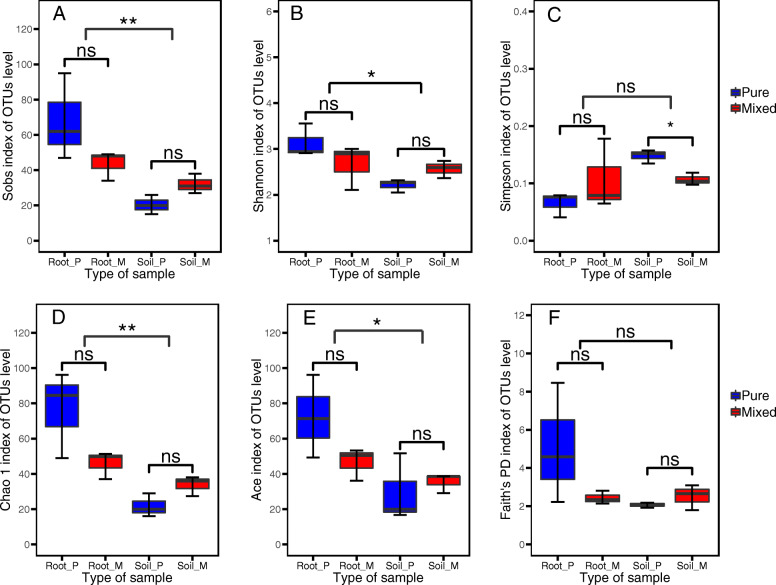
Alpha diversity indices of AM fungal communities in root and soil samples. For alpha diversity, number of OTUs observed (Sobs), Shannon, Simpson, Chao1, ACE and Faith’s PD indices were calculated using random subsamples of 15,869 18S rRNA gene sequences per sample. OTUs were delineated at 97% sequence similarity. The box plot shows median (black line), first quartile–third quartile percentiles (box range) and 1.5× the interquartile range (whiskers). There were three independent replicates of each treatment

The number of Sobs and the Shannon, Chao1, Ace and Faith PD indices of the root samples were higher in the pure plantation (Fig. 3a-f; *P* > 0.05), while the Sobs, Shannon, Chao1 and Faith PD indices of the soil samples were higher in the mixed plantation (*P* > 0.05). The Simpson index of the soil samples was significantly higher in the pure plantation than in the mixed plantation (*P* < 0.05). In the same type of sample (root or soil), the forest type did not have a significant effect on AM fungal diversity, but the Sobs, Shannon, Chao1 and Ace indices of AM fungi in the roots were significantly higher than those in the soil (*P* < 0.05).

**Fig. 4:**
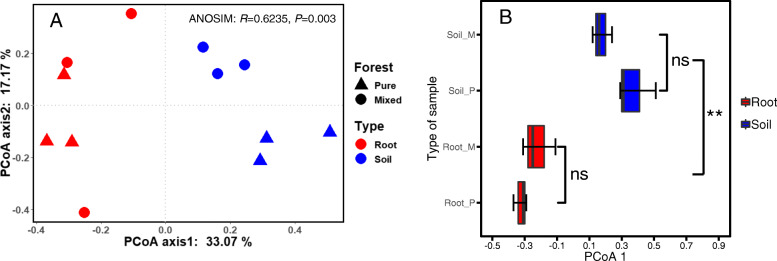
PCoA analyses of AM fungal communities in the root and soil samples. The PCoA plot was based on the Bray-Curtis distances at the OTU level (97% sequence similarity) of AM fungal communities

Principal coordinates analysis (PCoA) analysis at the OTU level showed that the samples from Root_P, Root_M, Soil_P and Soil_M were separated from each other (Fig. 4a). The two principal component axes explained more than 50% of the total variation (PCoA 1 = 33.07%, PCoA 2 = 17.17%). The analysis of similarities (ANOSIM) and permutation multivariate analysis of variance (PERMANOVA) demonstrated that the AM fungal community structure differed by sample type (*P* = 0.002) (Fig. 4b, Table S3).

### Shared and unique OTUs

**Fig. 5:**
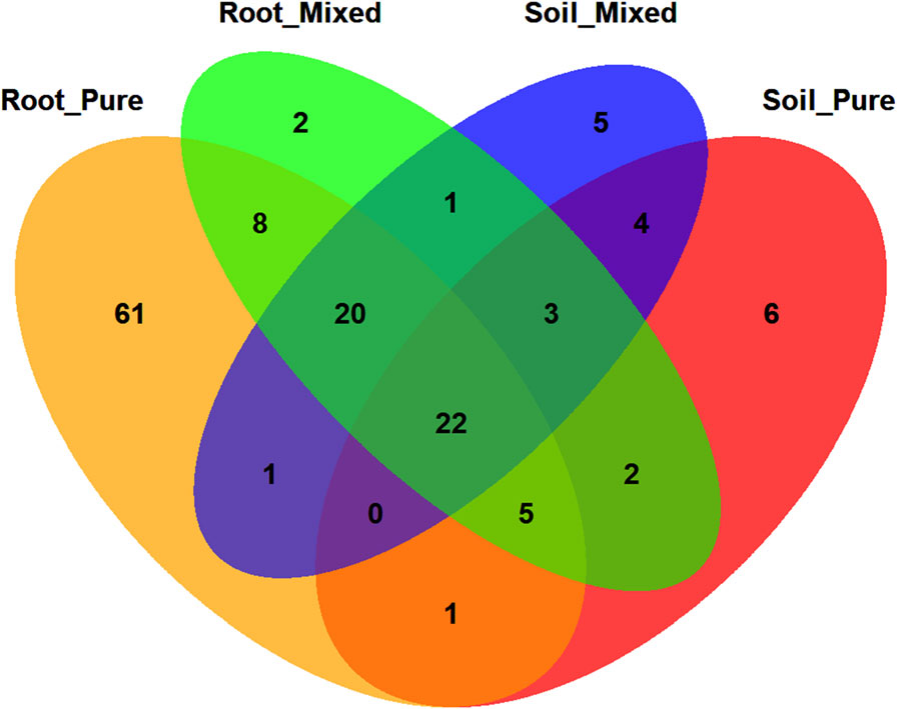
Venn diagram analyses of AM fungal communities in the root and soil samples. Venn diagram demonstrated the numbers of shared and unique observed OTUs at 97% similarity among treatments

**Fig. 6:**
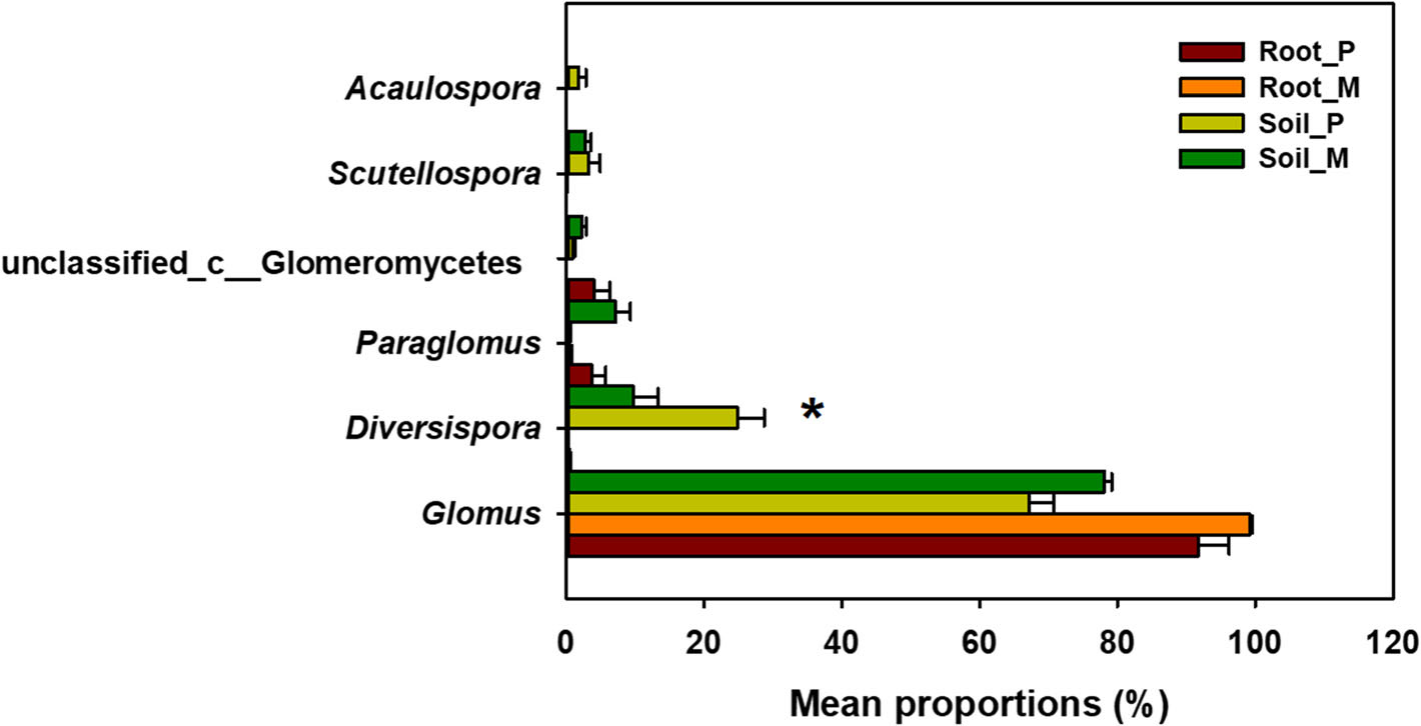
Relative abundances of main AM fungal genus in the root and soil samples. Genus and OTUs with average relative abundances > 1% were shown in at least one treatment. Values in the bar plot are expressed as mean ± standard error. Asterisks indicate significant difference between treatments based on Tukey’s test (*P* < 0.05)

The Venn diagram analysis showed that all samples shared 22 OTUs, which accounted for 7.86% of the total OTUs observed (Fig. 5). At the genus level, these shared OTUs mainly belonged to *Glomus*, *Paraglomus* and *Diversispora* (Table S4). Samples from Root_P had the highest number of unique OTUs, which mainly belonged to the unidentified genus of Glomeromycetes, *Paraglomus*, *Glomus* and *Acaulospora* (Table S5). The amount of unique OTUs detected in the soil was lower than those in the roots. At the genus level, Soil_P had a significantly higher relative abundance of *Diversispora* than the other treatments (Fig. 6, *P* < 0.05).

### Relationships between AM fungal communities and soil characteristics

**Fig. 7:**
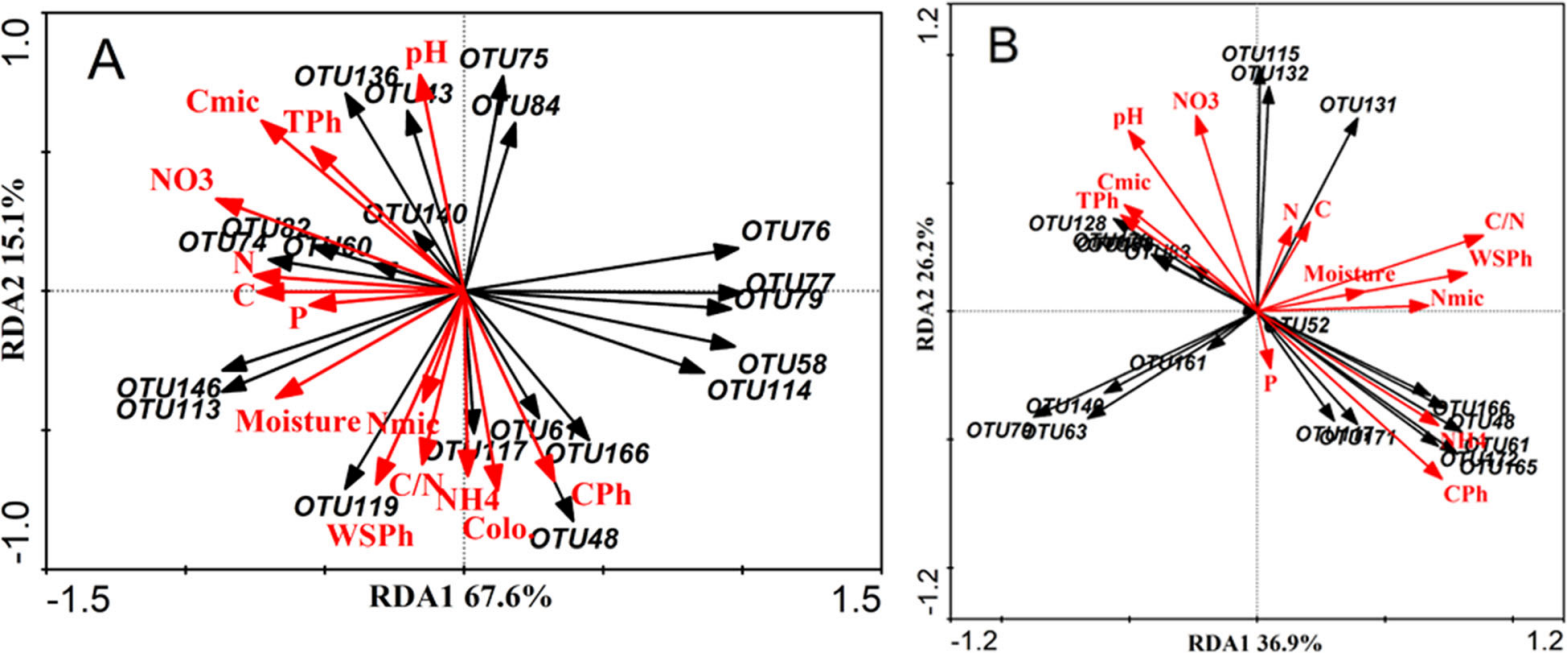
Ordination plots of the results from the redundancy analysis (RDA) to identify the relationships among the AM fungal taxa (Black arrows) and the soil characteristics (Red arrows). The top 20 most abundant classified AM fungal OTUs (97% sequence similarity) in the root (A) and soil (B) samples. C_mic_: soil microbial biomass carbon. N_mic_: soil microbial biomass nitrogen. TPh: soil total phenol. CPh: soil complex phenol. WSPh: soil water-soluble phenol. NO3: NO_3_^−^-N. NH4: NH_4_^+^-N

RDA and the Mantel test were conducted to identify the key drivers of AM fungal community structure. In the RDA plots of the AM fungal community structure in both plantations, soil NO_3_^−^-N, C_mic_ and pH appeared to be the most important soil characteristics controlling the root AM fungal community structure and showed major differences among microbial communities (Fig. 7a). The Mantel test demonstrated that root AM fungal abundance was significantly correlated with NO_3_^−^N (*R*^*2*^ = 0.906, *P* = 0.029) and C_mic_ (*R*^*2*^ = 0.881, *P* = 0.044) (Table 2). The soil complex phenols (CPh), C/N, pH, NO_3_^−^-N and NH_4_^+^-N had longer arrows than the others (Fig. 7b). This indicates that these variables have stronger impacts than others on the AM fungal community. Of all the soil characteristics tested, C/N (*R*^*2*^ = 0.864, *P* = 0.032) and CPh (*R*^*2*^ = 0.994, *P* = 0.019) were significantly correlated with the relative abundance of OTUs (Table 2).

**Table 2:** Mantel analysis on the relationship between the relative abundance of OTUs and soil or plant variables

Environmental variables	Root		Soil	
	*R*^*2*^	*P*	*R*^*2*^	*P*
pH	0.647	0.213	0.695	0.158
Moisture (%)	0.61	0.257	0.178	0.743
C (g·kg^−1^)	0.543	0.29	0.148	0.788
N (g·kg^−1^)	0.565	0.254	0.116	0.821
P (g·kg^−1^)	0.311	0.594	0.047	0.922
C/N	0.386	0.472	**0.864**	0.032*
C_mic_ (mg·kg^−1^)	**0.881**	0.044*	0.455	0.415
N_mic_ (mg·kg^−1^)	0.170	0.749	0.439	0.411
NH_4_^+^-N (mg·kg^−1^)	0.454	0.394	0.688	0.165
NO_3_^−^-N (mg·kg^−1^)	**0.906**	0.029*	0.656	0.208
TPh (mg·kg^−1^)	0.542	0.336	0.442	0.419
CPh (mg·kg^−1^)	0.603	0.257	**0.944**	0.019*
WSPh (mg·kg^−1^)	0.601	0.233	0.676	0.179
Colonization (%)	0.521	0.306	–	–

*C*_*mic*_ soil microbial biomass carbon, *N*_*mic*_ soil microbial biomass nitrogen, *TPh* soil total phenol, *CPh* soil complex phenol, *WSPh* soil water-souble phenol

## Discussion

### Response of AM fungal diversity, composition and community structure to mixed planting

The core species composition of AM fungal communities was very similar and conservative between the pure and mixed plantations (*Glomus* mainly, Fig. 1), which is consistent with the result of Senés-Guerrero and Schüßler [34]. They found that there is a conservative core species of AM fungi both at different stages of plant development and under different environmental conditions in Andean ecosystems. Plant diversity has been shown to increase with increasing mycorrhizal fungal diversity, and the diversity and species composition of plant communities also exert a reciprocal influence on their associated mycorrhizal communities [15]. Van der Heijden et al. [35] believed that the change in host plants caused by colonization with a single mycorrhizal species was an important factor determining the species composition and diversity of plant communities. However, some researchers have reported that there is a negative correlation between plant diversity and AM fungal community diversity [36]. The lower diversity of AM fungi in the mixed plantation than in the pure plantation may have been attributable to the lower “carrying capacity” in the mixed plantation, i.e., the lower number of walnut roots. In mixture, larch roots have greater plasticity in traits related to resource uptake than walnut roots [37]. In addition, larch root exudates alleviate the autotoxic effect caused by juglone, which is secreted by Manchurian walnut in mixed plantings. Our previous studies have found that larch root exudates promote changes in the soil microbial communities of Manchurian walnut and increase soil invertase and urease activities [38]. Salahuddin et al. [37] indicated that the ratio of root tip tissue in Manchurian walnut significantly increased after larch was introduced, but the mycorrhizal infection rate was decreased. Achatz et al. [39] found that mycorrhizal mycelia promoted the migration and transport of juglone secreted by plant roots and that mycorrhizal mycelia enhanced interspecific interactions through the study of species of the genus *Juglans* (Juglandaceae). Mummey and Rillig [20] found that spotted knapweed, an invasive plant, could significantly change the AM fungal community in the invasion site.

The AM fungal community composition and abundance were obviously different between the pure and mixed plantations (Fig. 2 and Fig. 6), and there were more OTUs in the soil of the mixed plantation (Fig. 5). The reasons may be as follows: (1) Compared with the pure plantation, the mixed plantation had more advantages in terms of litter quantity and quality, soil nutrients and stand structure. The consequent changes in the soil microenvironment led to variations in the AM fungal community and (2) larch affects the root system of Manchurian walnut when they are planted in mixture, which reshapes the mycorrhizal network. Chen et al. [40] found that abiotic factors (soil moisture content, nitrate and soil enzymes, etc.) had a greater impact on the soil AM fungal community when *Robinia pseudoacacia* was mixed with *Platycladus orientalis*. The growth of some plants may change the soil quality or other abiotic characteristics, which may lead to changes in rhizosphere AM fungal communities [41].

### Comparison of AM fungal communities in root and soil samples

Our study found that the AM fungal community in the root system of Manchurian walnut was significantly different from that in the rhizosphere soil, which is consistent with previous results from temperate steppe [42], temperate farmland [43] and Mediterranean shrub communities [44]. This is because plants select AM fungi to varying degrees based on their own nutritional requirements in different growth and development stages, and the selection inevitably leads to differences in the relative abundance, species and quantity of AM fungi in soil and roots. In addition, there are substantial differences between the environmental conditions of AM fungi in plant roots and those in soil. The living environment of AM fungi in roots is mainly regulated by the physiological activities of individual plants, while AM fungi in soil are mainly affected by external environmental conditions.

Many scholars believe that the AM fungal community in soil represents a species pool and that plants can freely recruit certain species, suggesting that the AM fungal richness in soil is higher than that in the roots [45–48]. However, some scholars have found that the abundance of AM fungi in roots and soil is the same [49–51] or that AM fungi are more abundant in the root system than in the soil [52, 53]. In this study, the amount of OTUs in roots was higher than that in soil samples (in both the pure and mixed plantations), and a large proportion of AM fungi were detected in roots. There are many potential methodological and biological explanations for the differences in the AM fungal community, especially for the low number of OTUs detected in soil. First, the biomass of AM fungi in soil is an order of magnitude lower than that in roots [54]. Therefore, the concentration of soil AM fungal DNA is relatively low. The DNA extracted from the roots is an accurate measure of the root subsample, while the very small mass extracted from the soils likely excludes numerous species that were present in the composite soil sample from which the subsample was removed [55].

Second, there are differences in the distribution of AM fungi of different taxa between the root (intraradical) and soil (extraradical) samples [56]. Glomeraceae spend fewer resources on their external structures than on their internal structures [57]. In this study, *Glomus* was more abundant in the roots than in the soil, which was consistent with previous research results. Some studies have found that Gigasporaceae and Acaulosporaceae many more external hyphae than internal structures. In this study, Diversisporaceae and Gigasporaceae were mainly found in soil, with low sequence numbers in the root system; this is consistent with earlier findings that Diversisporaceae and Gigasporaceae are poor root colonizers [42, 53, 57]. Paraglomeraceae was mainly found in soil samples, with only a few sequences in the roots, which is consistent with previous studies [42, 53].

## Conclusions

Our study showed that long-term (almost 30 years) mixed management had little effect on the AM fungal community in the roots of *Juglans mandshurica* and significantly changed the community composition, but not the diversity, of AM fungi in the soils. Samples from the roots in the pure plantation had the highest number of unique OTUs. The core species composition of AM fungal communities was very similar and conservative between the pure and mixed plantations. In the future, the combination of root traits and mycorrhizal symbiosis should be considered to comprehensively evaluate the mechanisms of nutrient absorption and utilization by mixed tree species to lay a foundation for sustainable plantation management.

## Methods

### Study area and sample design

The study site was located at the Maoershan Forest Research Station (127°30′–127°34′E, 45°21′–45°25′N) of Northeast Forestry University, Heilongjiang Province, China. This area is characterized by a continental monsoon climate with a windy spring, a warm and humid summer, and a dry and cold winter. The mean annual temperature is 2.8 °C, with the minimum temperature in January (− 40.9 °C) and the maximum temperature in July (34.2 °C). The frost-free period fluctuates between 120 and 140 days. The annual precipitation ranges from 600 to 800 mm. Soils are Hap-Boric Luvisols [58] with high organic matter content and well-developed horizons and are well drained.

In spring 1987, one-year-old seedlings of *Juglans mandshurica* and *Larix gmelinii* were obtained from the Maoershan Forest Farm and transplanted to the experimental station to establish the monoculture (*Juglans mandshurica*, JM) and mixed (*Juglans mandshurica* × *Larix gmelinii*, J × L) plantations of Manchurian walnut (each pure or mixed forest of ∼0.5 ha). The seedlings were planted in a 1.5 m × 1.5 m grid in each plantation and mixed in rows (three rows of JM × five rows of larch) in the mixed plantation. The plantations both have similar site conditions and an average gradient of 7°. Detailed tree growth information for each plantation is shown in Table S1. Voucher specimens of *J. mandshurica* and *L. gmelinii* were not deposited in this study since they are the most common trees in Northeast China.

### Sample collection

In April 2016, three random sampling plots (20 m × 30 m 0.06 ha) were selected from the pure and mixed plantations described above and were identified to serve as replicates. The distance between plots ranged from 350 m to 700 m. Rhizosphere soil and plant root samples were collected in July 2016. In each of these experimental plots, root samples were collected from nine individuals of *Juglans mandshurica* and mixed to form a composite sample from each plot. The rhizosphere soils were sampled in the 0–10 cm soil layer adjacent to the roots, and the sample soil was brushed off of the plant root systems. The soil and root samples were packed in an ice box and transported to the laboratory. Soil samples were sieved (1 mm mesh) to remove roots and debris, and subsamples were stored at − 80 °C for DNA extraction. The first three root orders of the roots of *Juglans mandshurica* are infected by mycorrhiza [59]. Root samples were washed using distilled water to remove soil particles and were stored at − 80 °C for DNA extraction.

### DNA extraction and PCR amplification

Microbial DNA was extracted from root and soil samples using the E.Z.N.A.® soil DNA Kit (Omega Biotek, Norcross, GA, U.S.) according to the manufacturer’s protocols. The final DNA concentration and purification were determined by a NanoDrop 2000 UV-vis spectrophotometer (Thermo Scientific, Wilmington, USA), and DNA quality was checked by 1% agarose gel electrophoresis. The fungal 18S rRNA genes were amplified by a nested PCR. According to the study of Lumini et al. 2010 [60], AML1 (5′-ATCAACTTTCGATGGTAGGATAGA-3′) and AML2 (5′-GAACCCAAACA CTTTGGTTTCC-3′), where the barcode is an eight-base sequence unique to each sample, were used in the first round of PCR, and AMV4-5NF (5′-AAGCTCGTAGTTGAATTTCG-3′) and AMDGR (5′-CCCAACTATCCCTATTAATCAT-3′) were used in the second round of PCR with a thermocycler PCR system (GeneAmp 9700, ABI, USA). The two rounds of PCR yielded amplicons of approximately 800 bp and 300 bp, respectively. PCRs were performed in triplicate in a 20 μL mixture containing 4 μL of 5 × FastPfu Buffer, 2 μL of 2.5 mM dNTPs, 0.8 μL of each primer (5 μM), 0.4 μL of FastPfu Polymerase and 10 ng of template DNA. The PCRs were conducted using the following program: 3 min of denaturation at 95 °C, 30 cycles of 30 s at 95 °C, 30 s for annealing at 55 °C, and 45 s for elongation at 72 °C, and a final extension at 72 °C for 10 min. The procedure for the second round of PCRs was the same as that for the first round, except for the cycle number, which was 35 [61].

### Illumina MiSeq sequencing

The resulting PCR products were extracted from a 2% agarose gel, further purified using the AxyPrep DNA Gel Extraction Kit (Axygen Biosciences, Union City, CA, USA) and quantified using QuantiFluor™-ST (Promega, USA) according to the manufacturer’s protocol. Purified amplicons were pooled in equimolar amounts and paired-end sequenced (2 × 250) on an Illumina MiSeq platform (Illumina, San Diego, USA) according to the standard protocols by Majorbio Bio-Pharm Technology Co., Ltd. (Shanghai, China). The raw reads were deposited into the NCBI Sequence Read Archive (SRA) database (Accession Number: SRP227587).

### Processing of sequencing data

Raw fastq files were demultiplexed, quality filtered by Trimmomatic and merged by FLASH with the following criteria [62]: (i) The reads were truncated at any site receiving an average quality score < 20 over a 50 bp sliding window. (ii) Primers were exactly matched, allowing two nucleotide mismatches, and reads containing ambiguous bases were removed. (iii) Sequences whose overlap was longer than 10 bp were merged according to their overlap sequence.

Operational taxonomic units (OTUs) were clustered with a 97% similarity cutoff using UPARSE (version 7.1 http://drive5.com/uparse/) [63], and chimeric sequences were identified and removed using UCHIME. The taxonomy of each 18S rRNA gene sequence was analyzed by BLAST against the MaarjAM database with a confidence threshold of 70%.

### Soil physicochemical analyses

Total carbon (C) and total nitrogen (N) were measured by a Macro Elemental Analyzer (vario MACRO, Elementar Co., Germany), and total phosphorus (P) was determined colorimetrically with a UV spectrophotometer (TU-1901, Puxi Ltd., Beijing, China) after wet digestion with HClO_4_–H_2_SO_4_. Soil pH was measured using a pH meter (MT-5000, Shanghai). Soil nitrate-N (NO_3_^−^-N) and ammonium-N (NH_4_^+^-N) were extracted in 2 M KCl and measured using a continuous-flow ion autoanalyzer (Scalar SANplus segmented flow analyzer, The Netherlands). The soil total phenol content was measured by the ultraviolet spectrophotometer method [64]. The Folin reagent colorimetric method was used to determine soil water-soluble phenol and complex phenol content [64]. Soil microbial biomass carbon (MBC) and nitrogen (MBN) were measured using a chloroform fumigation extraction method [65]. The mycorrhizal colonization rate of fine roots was determined according to the method of Guo [59].

### Statistical analysis

For the Illumina MiSeq sequencing data, the alpha diversity indices (number of observed OTUs, Chao1, ACE, Faith’s PD, Shannon and Simpson diversity indices) were generated using QIIME [66]. For the beta diversity analysis, Bray-Curtis distances were calculated, and principal coordinate analysis (PCoA) was conducted to visualize the community similarity using the ‘vegan’ package in ‘R’ (Version 3.6.1) [67]. Analysis of similarities (ANOSIM) and permutation multivariate analysis of variance (PERMANOVA) were carried out to test the differences among microbial communities with the Bray-Curtis distances and 999 permutations. Heat map analysis was used to compare the relative abundances of the top 50 most abundant classified AM fungal genera among treatments with the ‘pheatmap’ package in ‘R’ (Version 3.6.1). The shared and unique OTUs among treatments were counted, and their distributions are shown in a Venn diagram created with the ‘VennDiagram’ package in ‘R’ (Version 3.6.1). Differences in the relative abundance of microbial taxa between treatments were analyzed using Welch’s *t* test and Tukey’s honestly significant difference (HSD) test with Bonferroni correction in ‘STAMP’ [68]. The differences were considered statistically significant if *P* < 0.05. Redundancy analysis (RDA) was used to identify the soil properties that predicted the variations in the AM fungal communities. The Mantel test with a Monte Carlo simulation with 999 randomizations was used to assess the relationships between the Euclidean distance of the AM fungal community and the soil characteristics. RDA and Mantel test analyses were performed with the rda function in the ‘vegan’ package and the mantel.rtest function in the ‘ade4’ package in ‘R’ (Version 3.6.1), respectively.

## Supplementary information

**Additional file 1: Figure S1.** The Rarefaction curves of the number of operational taxonomic units (OTUs) for AM fungal communities. **Figure S2.** Relative abundances of main AM fungal genus (A, C, E, G) and OTUs (B, D, F, H) in the root and soil samples. **Table S1.** Summary of stand and soil characteristics of three plantations used in this study: pure plantation of *Juglans mandshurica* and mixed plantation of *Juglans mandshurica* × *Larix gmelinii* in NE China. **Table S2.** Relative abundances (%) of the top 50 most abundant classified arbuscular mycorrhizal fungal OTUs in the root and soil samples. **Table S3.** Dissimilarity analysis of AM fungal communities with permutation multivariate analysis of variance (PERMANOVA). **Table S4.** Sequence of OTUs all shared to the root and soil samples in pure and mixed plantation. **Table S5.** Sequence of OTUs all unique to the root samples in pure plantation.

## Data Availability

All datasets are presented in the main text and the additional file. The raw sequence data on 18S rRNA gene amplicons have been submitted to the NCBI Sequence Read Archive (SRA) database (Accession Number: SRP227587). The dataset analyzed during the current study is available from the corresponding author on reasonable request.

## Abbreviations

AM: Arbuscular mycorrhiza
OTUs: Operational Taxonomic Units
C_mic_: Soil microbial biomass carbon
N_mic_: Soil microbial biomass nitrogen
TPh: Soil total phenol
CPh: Soil complex phenol
WSPh: Soil water-souble phenol
Soil_P: Soil sample in pure plantation
Soil_M: Soil sample in mixed plantation
Root_P: Root sample in pure plantation
Root_M: Root sample in mixed plantation
