# Supplementary material for: Long-term effects of mixed planting on arbuscular mycorrhizal fungal communities in the roots and soils of Juglans mandshurica plantations
Source: BMC Microbiol. 2020 Oct 12;20:304. doi: 10.1186/s12866-020-01987-1 (PMC7552469; doi:10.1186/s12866-020-01987-1)
Supplement: Supplementary file 1 — Additional file 1: Figure S1. The Rarefaction curves of the number of operational taxonomic units (OTUs) for AM fungal communities. Figure S2. Relative abundances of main AM fungal genus (A, C, E, G) and OTUs (B, D, F, H) in the root and soil samples. Table S1. Summary of stand and soil characteristics of three plantations used in this study: pure plantation of Juglans mandshurica and mixed plantation of Juglans mandshurica × Larix gmelinii in NE China. Table S2. Relative abundances (%) of the top 50 most abundant classified arbuscular mycorrhizal fungal OTUs in the root and soil samples. Table S3. Dissimilarity analysis of AM fungal communities with permutation multivariate analysis of variance (PERMANOVA). Table S4. Sequence of OTUs all shared to the root and soil samples in pure and mixed plantation. Table S5. Sequence of OTUs all unique to the root samples in pure plantation. [file 12866_2020_1987_MOESM1_ESM.docx]

**Long-term Effects of Mixed Management on Arbuscular Mycorrhizal Fungal Community of Root and Soil in *Juglans mandshurica* Plantation**

**Li Ji^1,2^, Yan Zhang^1^, Lixue Yang^1*^, Yuchun Yang^2*^, Na Yang^1,3^, Depeng Zhang^1^**

^1^ *Key Laboratory of Sustainable Forest Ecosystem Management-Ministry of Education, School of Forestry, Northeast Forestry University, Harbin 150040, P. R. China*

^2^ *Jilin Academy of Forestry, Changchun 130033, P.R. China*

*^3^* *ZEHO* *Waterfront Ecological Environment Management Co., Ltd., Beijing 100084, P. R. China*

****Correspondence:***

**Lixue Yang ylx_0813@163.com**

**Yuchun Yang yang-yu-chun@163.com**

**Supplementary data is AM fungal taxonomic data, please see xls. file.**

**Figure captions:**

**Fig. S1 The Rarefaction curves of the number of operational taxonomic units (OTUs) for AM fungal communities.** Random subsamples of 15,869 18S rRNA gene per sample were used to generate the rarefaction curves. OTUs were delineated at 97% sequence similarity. Root_Pure, Root_Mixed, Soil_Pure and Soil_Mixed represent root and soil samples of Manchurian walnut of pure and mixed plantations, respectively.

**Fig. S2 Relative abundances of main AM fungal genus (A, C, E, G) and OTUs (B, D, F, H) in the root and soil samples.** Genus and OTUs with average relative abundances > 1% were shown in at least one treatment. Values in the bar plot are expressed as mean ± standard error. The colored circles represent the 95% confidence intervals. Asterisks indicate significant difference between treatments based on Welch’s *t* test (*P* < 0.05)

**Fig. S1**


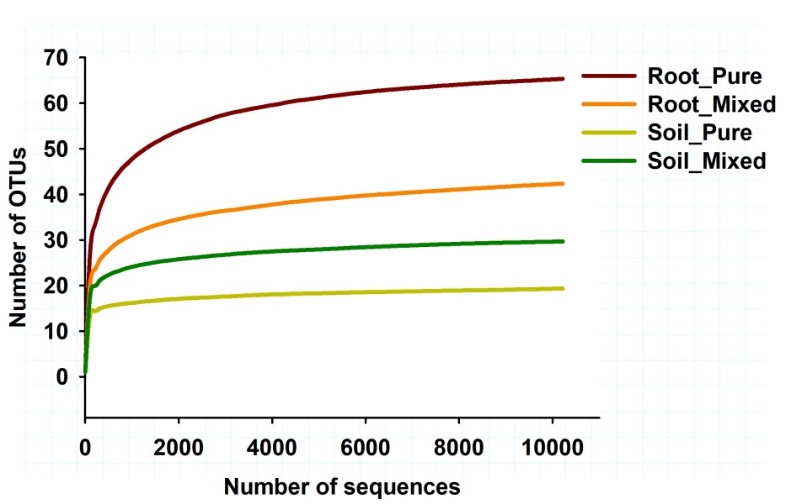


**Fig. S2**

**
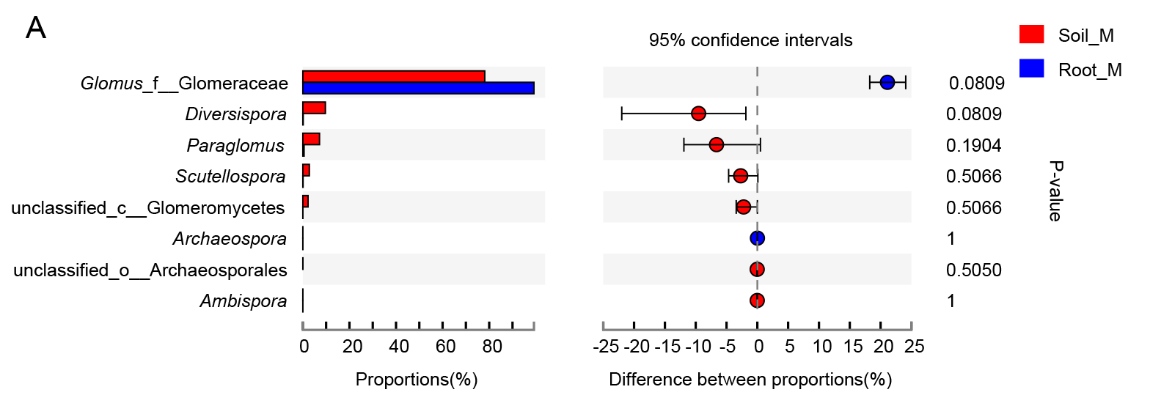

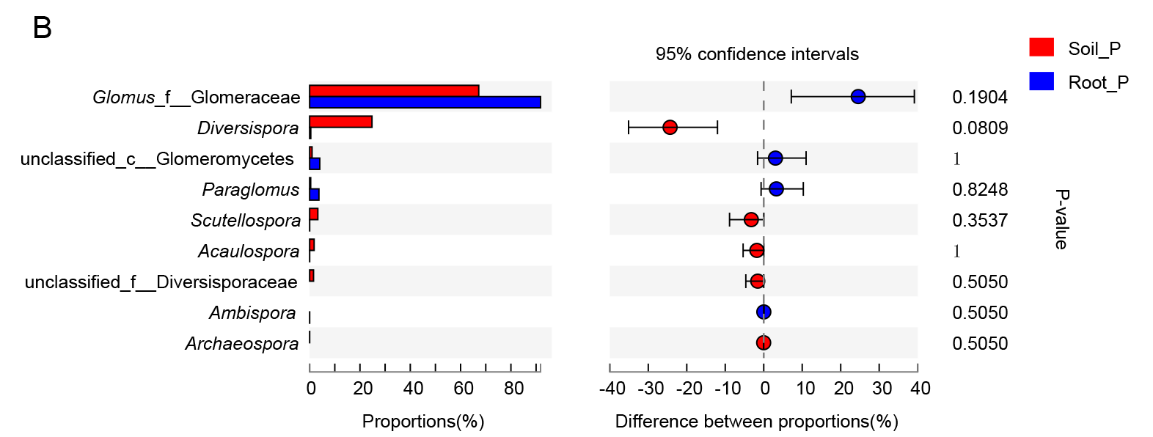

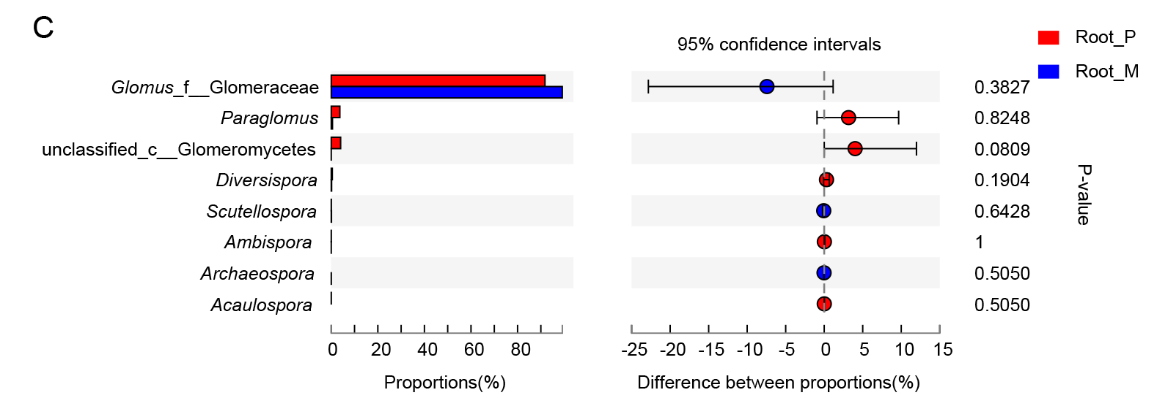

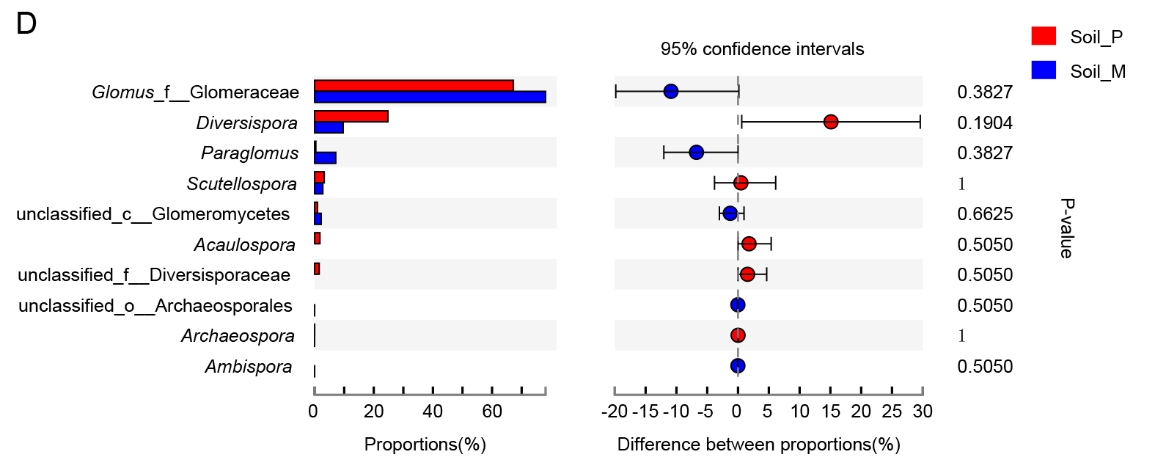
**

**Table S1 Summary of stand and soil characteristics of three plantations used in this study: pure plantation of *Juglans mandshurica* and mixed plantation of *Juglans mandshurica* × *Larix gmelinii* in NE China.** Stand characteristics given are tree density, diameter at breast height (DBH) and tree height, soil characteristics (0–10 cm soil depth without litter) given are pH (H_2_O), organic matter (OM), total and hydrolytic Nitrogen (N), and total and available Phosphorous (P) (mean; n_DBH/Height_= 13–15, n_Soil_ = 3–5).

| Plantation type | Species | Stand characteristics | | | Soil characteristics | | | | | |
| --- | --- | --- | --- | --- | --- | --- | --- | --- | --- | --- |
|  |  | Density  (n ha^-1^) | DBH  (cm) | Height  (m) | pH | OM  (%) | Total N  (g kg^-1^) | Hydrolytic N  (mg kg^-1^) | Total P  (g kg^-1^) | Available P  (mg kg-1) |
| Pure | *Juglans mandshurica* | 1500 | 54.9 | 11.8 | 4.9 | 6.63 | 5.72 | 451.3 | 0.59 | 7.5 |
| Mixed | Average | 1380 | —— | —— | 5.1 | 6.41 | 6.47 | 524.1 | 0.61 | 8.5 |
| Mixed | *Juglans mandshurica* | 645 | 65.4 | 13.0 | —— | —— | —— | —— | —— | —— |
| Mixed | *Larix gmelinii* | 735 | 41.7 | 13.7 | —— | —— | —— | —— | —— | —— |

**Table S2 Relative abundances (%) of the top 50 most abundant classified arbuscular mycorrhizal fungal OTUs in the root and soil samples.** Values (mean±SE) with different letters are significantly different at the 0.05 probability level (Kruskal Wallis test).

| OTU ID | Root_Pure | Root_Mixed | Soil_Pure | Soil_Mixed | OTU ID | Root_Pure | Root_Mixed | Soil_Pure | Soil_Mixed |
| --- | --- | --- | --- | --- | --- | --- | --- | --- | --- |
| OTU79 | 2.88±0.92b | 5.17±2.98b | 23.13±5.3a | 17.41±4.01a | OTU55 | —— | 1.41±0.81a | 3.61±3.61a | —— |
| OTU76 | 10.05±4.42a | 12.8±9.51a | 0.04±0.04a | 3.25±2.96a | OTU176 | 0.09±0.05a | 1.5±1.5a | 2.4±2.4a | 0.5±0.5a |
| OTU128 | 0.38±0.14ab | 0.15±0.14b | 14.02±3.66a | 9.5±6.4a | OTU60 | 2.82±1.42a | 1.13±0.55a | —— | 0.51±0.48a |
| OTU58 | 8.32±2.05ab | 12.36±5.69a | 1.14±1.14b | 1.11±0.45b | OTU161 | 0.03±0.03a | 0.17±0.17a | 0.62±0.62a | 3.22±2.39a |
| OTU48 | 0.67±0.16a | 7.22±2.39a | —— | 6.88±6.58a | OTU63 | 0.32±0.27a | 0.44±0.43a | 3.22±3.22a | —— |
| OTU61 | 4.62±1.1a | 4.37±1.92a | 0.01±0.01b | 5.27±1.21a | OTU52 | 0.78±0.35a | 0.15±0.12a | 1.79±1.79a | 1.11±1.1a |
| OTU113 | 5.64±2.61a | 6.36±4.09a | 1.06±1.06a | 0.43±0.43a | OTU77 | 1.53±0.49a | 2.24±1.49a | —— | —— |
| OTU43 | 10.52±3.68a | 1.14±0.87b | —— | 1.36±1.36b | OTU75 | 3.46±3.04a | 0.28±0.28a | —— | —— |
| OTU166 | 2.19±1.15a | 3.59±2.03a | —— | 6.16±2.69a | OTU151 | 2.24±1.57a | 1.18±0.62a | —— | 0.05±0.03a |
| OTU117 | 3.09±0.48ab | 2.9±1.57ab | 0.27±0.27b | 5.66±1.73a | OTU84 | 2.86±2.86a | 0.6±0.6a | —— | —— |
| OTU140 | 2.2±1.15a | 2.5±1.22a | 6.25±4.63a | 0.48±0.48a | OTU81 | —— | 0.08±0.07a | 0.29±0.29a | 2.79±1.44a |
| OTU74 | 3.8±2.08a | 4.45±4.38a | 2.19±2.19a | 0.06±0.06a | OTU143 | 0.47±0.47a | 0.06±0.05a | —— | 2.53±1.66a |
| OTU115 | 0.64±0.51ab | 0.26±0.2b | 7.44±4.16a | 2.01±0.48ab | OTU178 | —— | 0.01±0.01a | 2.97±2.97a | —— |
| OTU132 | —— | —— | 9.37±7.13a | 0.18±0.18a | OTU158 | 0.49±0.38a | 0.88±0.48a | —— | 1.08±0.57a |
| OTU136 | 5.17±1.3a | 3.27±2.45ab | 0.35±0.35b | #VALUE! | OTU44 | 0.06±0.04b | 1.77±0.53a | —— | 0.51±0.51b |
| OTU114 | 2.16±1.3a | 4.07±2.51a | 1.45±0.69a | 0.98±0.98a | OTU141 | 1.25±1.23a | 0.27±0.25a | 0.73±0.73a | —— |
| OTU131 | 0.04±0.04a | 0.39±0.39a | 5.99±5.99a | 1.53±1.53a | OTU129 | —— | —— | 1.79±1.79a | —— |
| OTU119 | 1.66±0.52a | 5.19±2.58a | —— | 0.87±0.87a | OTU39 | 1.73±1.57a | —— | —— | —— |
| OTU165 | —— | 0.49±0.48b | 0.16±0.14b | 6.86±3.59a | OTU123 | —— | —— | 1.55±1.55a | —— |
| OTU146 | 2.29±1.01a | 2.66±1.83a | 2.1±2.1a | —— | OTU50 | 1.51±0.8a | —— | —— | —— |
| OTU82 | 1.81±1.02a | 4.2±4.19a | —— | 0.4±0.4a | OTU142 | 0.07±0.07b | —— | 1.34±0.32a | —— |
| OTU172 | —— | 0.06±0.06b | 0.72±0.72b | 5.27±2.64a | OTU51 | 1.21±0.7a | 0.13±0.12ab | —— | 0.02±0.02b |
| OTU171 | 0.64±0.14a | 0.38±0.38a | —— | 4.96±3.7a | OTU139 | —— | —— | 0.27±0.27a | 0.95±0.95a |
| OTU83 | 0.73±0.71a | 0.99±0.89a | 1.43±1.43a | 2.76±2.76a | OTU153 | —— | —— | —— | 1.06±1.06a |
| OTU167 | 1.96±0.51a | 1.75±0.78a | 0.6±0.6a | 0.87±0.85a | OTU170 | 0.42±0.42a | 0.05±0.05a | 0.25±0.25a | 0.3±0.17a |

Values (mean± SE) with diﬀerent letters are significantly diﬀerent at the 0.05 probability level (Tukey’s HSD test).

**Table S3 Dissimilarity analysis of AM fungal communities with permutation multivariate analysis of variance (PERMANOVA).**

|  | Df | SumsOfSqs | MeanSqs | F.Model | *R^2^* | Pr(>F) |
| --- | --- | --- | --- | --- | --- | --- |
| Forest type & types of samples | 3 | 1.4603 | 0.48675 | 2.366 | 0.47013 | 0.002 |
| Residuals | 8 | 1.6458 | 0.20573 |  | 0.52987 |  |
| Total | 11 | 3.1061 |  |  | 1 |  |

**Table S4 Sequence of OTUs all shared to the root and soil samples in pure and mixed plantation.**

| Number | Order | Family | Genus | OTU ID |
| --- | --- | --- | --- | --- |
| 1 | Diversisporales | Diversisporaceae | *Diversispora* | OTU128 |
| 2 | Glomerales | Glomeraceae | *Glomus* | OTU117 |
| 3 | Glomerales | Glomeraceae | *Glomus* | OTU79 |
| 4 | Glomerales | Glomeraceae | *Glomus* | OTU74 |
| 5 | Glomerales | Glomeraceae | *Glomus* | OTU76 |
| 6 | Glomerales | Glomeraceae | *Glomus* | OTU172 |
| 7 | Glomerales | Glomeraceae | *Glomus* | OTU61 |
| 8 | Glomerales | Glomeraceae | *Glomus* | OTU80 |
| 9 | Glomerales | Glomeraceae | *Glomus* | OTU58 |
| 10 | Glomerales | Glomeraceae | *Glomus* | OTU167 |
| 11 | Glomerales | Glomeraceae | *Glomus* | OTU115 |
| 12 | Glomerales | Glomeraceae | *Glomus* | OTU161 |
| 13 | Glomerales | Glomeraceae | *Glomus* | OTU45 |
| 14 | Glomerales | Glomeraceae | *Glomus* | OTU114 |
| 15 | Glomerales | Glomeraceae | *Glomus* | OTU43 |
| 16 | Glomerales | Glomeraceae | *Glomus* | OTU140 |
| 17 | Glomerales | Glomeraceae | *Glomus* | OTU131 |
| 18 | Glomerales | Glomeraceae | *Glomus* | OTU176 |
| 19 | Glomerales | Glomeraceae | *Glomus* | OTU83 |
| 20 | Glomerales | Glomeraceae | *Glomus* | OTU113 |
| 21 | Glomerales | Glomeraceae | *Glomus* | OTU52 |
| 22 | Paraglomerales | Paraglomeraceae | *Paraglomus* | OTU170 |

**Table S5 Sequence of OTUs all unique to the root samples in pure plantation.**

| Number | Order | Family | Genus | OTU ID |
| --- | --- | --- | --- | --- |
| 1 | Diversisporales | Gigasporaceae | *Scutellospora* | OTU31 |
| 2 | Diversisporales | Acaulosporaceae | *Acaulospora* | OTU71 |
| 3 | Glomerales | Glomeraceae | *Glomus* | OTU66 |
| 4 | Glomerales | Glomeraceae | *Glomus* | OTU37 |
| 5 | Glomerales | Glomeraceae | *Glomus* | OTU68 |
| 6 | Glomerales | Glomeraceae | *Glomus* | OTU147 |
| 7 | Glomerales | Glomeraceae | *Glomus* | OTU70 |
| 8 | Glomerales | Glomeraceae | *Glomus* | OTU73 |
| 9 | Glomerales | Glomeraceae | *Glomus* | OTU30 |
| 10 | Glomerales | Glomeraceae | *Glomus* | OTU8 |
| 11 | Glomerales | Glomeraceae | *Glomus* | OTU35 |
| 12 | Glomerales | Glomeraceae | *Glomus* | OTU62 |
| 13 | Glomerales | Glomeraceae | *Glomus* | OTU27 |
| 14 | Glomerales | Glomeraceae | *Glomus* | OTU21 |
| 15 | Glomerales | Glomeraceae | *Glomus* | OTU2 |
| 16 | Glomerales | Glomeraceae | *Glomus* | OTU3 |
| 17 | Glomerales | Glomeraceae | *Glomus* | OTU94 |
| 18 | Glomerales | Glomeraceae | *Glomus* | OTU36 |
| 19 | Glomerales | Glomeraceae | *Glomus* | OTU1 |
| 20 | Glomerales | Glomeraceae | *Glomus* | OTU49 |
| 21 | Glomerales | Glomeraceae | *Glomus* | OTU42 |
| 22 | Glomerales | Glomeraceae | *Glomus* | OTU14 |
| 23 | Glomerales | Glomeraceae | *Glomus* | OTU50 |
| 24 | Paraglomerales | Paraglomeraceae | *Paraglomus* | OTU72 |
| 25 | Paraglomerales | Paraglomeraceae | *Paraglomus* | OTU98 |
| 26 | Paraglomerales | Paraglomeraceae | *Paraglomus* | OTU39 |
| 27 | Paraglomerales | Paraglomeraceae | *Paraglomus* | OTU22 |
| 28 | unclassified_c__Glomeromycetes | unclassified_c__Glomeromycetes | unclassified_c__Glomeromycetes | OTU89 |
| 29 | unclassified_c__Glomeromycetes | unclassified_c__Glomeromycetes | unclassified_c__Glomeromycetes | OTU38 |
| 30 | unclassified_c__Glomeromycetes | unclassified_c__Glomeromycetes | unclassified_c__Glomeromycetes | OTU34 |
| 31 | unclassified_c__Glomeromycetes | unclassified_c__Glomeromycetes | unclassified_c__Glomeromycetes | OTU33 |
| 32 | unclassified_c__Glomeromycetes | unclassified_c__Glomeromycetes | unclassified_c__Glomeromycetes | OTU32 |
| 33 | unclassified_c__Glomeromycetes | unclassified_c__Glomeromycetes | unclassified_c__Glomeromycetes | OTU29 |
| 34 | unclassified_c__Glomeromycetes | unclassified_c__Glomeromycetes | unclassified_c__Glomeromycetes | OTU26 |
| 35 | unclassified_c__Glomeromycetes | unclassified_c__Glomeromycetes | unclassified_c__Glomeromycetes | OTU24 |
| 36 | unclassified_c__Glomeromycetes | unclassified_c__Glomeromycetes | unclassified_c__Glomeromycetes | OTU25 |
| 37 | unclassified_c__Glomeromycetes | unclassified_c__Glomeromycetes | unclassified_c__Glomeromycetes | OTU23 |
| 38 | unclassified_c__Glomeromycetes | unclassified_c__Glomeromycetes | unclassified_c__Glomeromycetes | OTU20 |
| 39 | unclassified_c__Glomeromycetes | unclassified_c__Glomeromycetes | unclassified_c__Glomeromycetes | OTU40 |
| 40 | unclassified_c__Glomeromycetes | unclassified_c__Glomeromycetes | unclassified_c__Glomeromycetes | OTU41 |
| 41 | unclassified_c__Glomeromycetes | unclassified_c__Glomeromycetes | unclassified_c__Glomeromycetes | OTU104 |
| 42 | unclassified_c__Glomeromycetes | unclassified_c__Glomeromycetes | unclassified_c__Glomeromycetes | OTU109 |
| 43 | unclassified_c__Glomeromycetes | unclassified_c__Glomeromycetes | unclassified_c__Glomeromycetes | OTU13 |
| 44 | unclassified_c__Glomeromycetes | unclassified_c__Glomeromycetes | unclassified_c__Glomeromycetes | OTU111 |
| 45 | unclassified_c__Glomeromycetes | unclassified_c__Glomeromycetes | unclassified_c__Glomeromycetes | OTU110 |
| 46 | unclassified_c__Glomeromycetes | unclassified_c__Glomeromycetes | unclassified_c__Glomeromycetes | OTU69 |
| 47 | unclassified_c__Glomeromycetes | unclassified_c__Glomeromycetes | unclassified_c__Glomeromycetes | OTU9 |
| 48 | unclassified_c__Glomeromycetes | unclassified_c__Glomeromycetes | unclassified_c__Glomeromycetes | OTU7 |
| 49 | unclassified_c__Glomeromycetes | unclassified_c__Glomeromycetes | unclassified_c__Glomeromycetes | OTU6 |
| 50 | unclassified_c__Glomeromycetes | unclassified_c__Glomeromycetes | unclassified_c__Glomeromycetes | OTU5 |
| 51 | unclassified_c__Glomeromycetes | unclassified_c__Glomeromycetes | unclassified_c__Glomeromycetes | OTU4 |
| 52 | unclassified_c__Glomeromycetes | unclassified_c__Glomeromycetes | unclassified_c__Glomeromycetes | OTU99 |
| 53 | unclassified_c__Glomeromycetes | unclassified_c__Glomeromycetes | unclassified_c__Glomeromycetes | OTU97 |
| 54 | unclassified_c__Glomeromycetes | unclassified_c__Glomeromycetes | unclassified_c__Glomeromycetes | OTU95 |
| 55 | unclassified_c__Glomeromycetes | unclassified_c__Glomeromycetes | unclassified_c__Glomeromycetes | OTU17 |
| 56 | unclassified_c__Glomeromycetes | unclassified_c__Glomeromycetes | unclassified_c__Glomeromycetes | OTU16 |
| 57 | unclassified_c__Glomeromycetes | unclassified_c__Glomeromycetes | unclassified_c__Glomeromycetes | OTU15 |
| 58 | unclassified_c__Glomeromycetes | unclassified_c__Glomeromycetes | unclassified_c__Glomeromycetes | OTU12 |
| 59 | unclassified_c__Glomeromycetes | unclassified_c__Glomeromycetes | unclassified_c__Glomeromycetes | OTU11 |
| 60 | unclassified_c__Glomeromycetes | unclassified_c__Glomeromycetes | unclassified_c__Glomeromycetes | OTU10 |
| 61 | unclassified_c__Glomeromycetes | unclassified_c__Glomeromycetes | unclassified_c__Glomeromycetes | OTU19 |
